# HDACs in the Brain: From Chromatin Remodeling to Neurodegenerative Disease

**DOI:** 10.3390/cells14171338

**Published:** 2025-08-29

**Authors:** Luan Pereira Diniz, Pedro de Sena Murteira Pinheiro, Lucas S. Franco, Flávia Carvalho Alcantara Gomes

**Affiliations:** 1 Laboratório de Investigação Metabólica Associada ao Envelhecimento, Instituto de Ciências Biomédicas, Universidade Federal do Rio de Janeiro, Rio de Janeiro 21941-902, RJ, Brazil; 2 Laboratório de Avaliação e Síntese de Substâncias Bioativas (LASSBio), Instituto de Ciências Biomédicas, Universidade Federal do Rio de Janeiro, Rio de Janeiro 21941-902, RJ, Brazil; 3 Laboratório de Neurobiologia Celular, Instituto de Ciências Biomédicas, Universidade Federal do Rio de Janeiro, Rio de Janeiro 21941-902, RJ, Brazil

**Keywords:** histone deacetylases, epigenetic regulation, neurogenesis, synaptic plasticity, glial cells, neuroinflammation, HDAC inhibitors, neurodegenerative diseases

## Abstract

Histone deacetylases (HDACs) are key epigenetic regulators that influence chromatin remodeling, gene expression, and cellular plasticity in the central nervous system (CNS). This review provides a comprehensive overview of the classification and functional diversity of HDACs, with particular emphasis on their roles in neural progenitor cells, mature neurons, and glial populations. In neural stem and progenitor cells, HDACs modulate neurogenesis, fate specification, and lineage commitment. In differentiated neurons, HDACs govern synaptic plasticity, memory formation, and survival. In glial cells, including astrocytes and microglia, HDACs orchestrate inflammatory responses, redox balance, and metabolic adaptations. We further examine the dysregulation of HDAC expression and activity in major neurodegenerative diseases, including Alzheimer’s disease and Parkinson’s disease. Evidence from human post-mortem brain studies reveals region- and isoform-specific alterations in HDAC expression, which are closely associated with cognitive decline, mitochondrial dysfunction, and neuroinflammation. Preclinical studies support the use of HDAC inhibitors (HDACi) as neuroprotective agents, capable of restoring acetylation homeostasis, reducing neuroinflammation, and improving neuronal function. Given the relevance of HDACi, we summarize current clinical studies assessing the safety of these compounds in the context of tumor biology, as well as their potential future applications in neurodegenerative diseases. Together, this review underscores the dual significance of HDACs as biomarkers and therapeutic targets in the context of CNS disorders.

## 1. Introduction

The global aging population is rapidly increasing, leading to a significant rise in the prevalence of neurodegenerative diseases (NDDs) such as Alzheimer’s disease (AD), Parkinson’s disease (PD), and Huntington’s disease (HD). These conditions, which primarily affect the central nervous system (CNS), are characterized by neuronal death, synaptic dysfunction, and neuroinflammation, leading to a decline in cognitive and motor abilities. Those illnesses represent a major challenge in medicine due to their complexity, multifactorial nature, and lack of efficient treatment [1]. These conditions not only severely impact the quality of life of individuals but also place an immense burden on public health systems due to the long-term care and treatment they require. The financial costs associated with managing these diseases are escalating, straining healthcare resources worldwide. Despite advances in understanding the pathophysiology of these diseases, effective therapies that halt or slow their progression remain elusive.

Emerging evidence on NDDs research has been shedding light on epigenetic regulators such as histone deacetylases (HDACs) as new targets. These enzymes play a pivotal role in regulating gene expression through chromatin remodeling, influencing processes like cell proliferation, differentiation, and survival. HDACs have gained attention as potential therapeutic targets for NDDs due to their involvement in neuronal plasticity, neuroinflammation, and neuronal survival [2]. HDAC inhibitors (HDACi), small molecules that prevent histone deacetylation, can promote a more relaxed chromatin structure, thus enhancing gene expression related to neuroprotective pathways. Preclinical and clinical studies have highlighted the neuroprotective potential of HDACi, demonstrating their ability to mitigate neurodegeneration, reduce neuroinflammation, and improve cognitive and motor functions [3].

Although the role of HDACi in neurons has been widely explored, recent findings suggest that glial cells, which play a critical role in the neurodegenerative process, are also a target for HDACi. Glial cells contribute significantly to neuroinflammatory responses, synaptic regulation, and the overall progression of NDDs. These cells, once considered supportive elements, are now recognized as active participants in disease pathology [4]. As a result, targeting astrocytic pathways with HDACi offers a novel and promising approach to modulate the neuroinflammatory environment, providing a complementary strategy to neuron-targeted therapies.

This review aims to explore the potential of HDACi in the treatment of NDDs, focusing on their molecular mechanisms and therapeutic benefits. It will also discuss the emerging role of astrocytes as cellular targets in HDACi-based therapies, highlighting how this new direction could enhance the efficacy of treatments for these debilitating conditions.

## 2. Classification and Functional Roles of Histone Deacetylases

HDACs are classified into four major classes based on their sequence homology to yeast counterparts, subcellular localization, and cofactor dependency. Class I HDACs (HDAC1, 2, 3, and 8) are primarily nuclear and ubiquitously expressed, playing essential roles in transcriptional repression, cell proliferation, and neurogenesis [5]. These isoforms are critical for maintaining chromatin structure and regulating gene expression programs necessary for neural development and synaptic plasticity. Class II HDACs are further subdivided into class IIa (HDAC4, 5, 7, and 9) and class IIb (HDAC6 and 10). Class IIa HDACs shuttle between the nucleus and cytoplasm and are involved in tissue-specific gene regulation, particularly in response to neural activity. Class IIb HDACs, notably HDAC6, localize mainly in the cytoplasm and regulate non-histone substrates such as α-tubulin, contributing to cytoskeletal dynamics, protein trafficking, and autophagy, processes relevant to neuronal integrity and neurodegeneration [6]. Class III HDACs, known as sirtuins (SIRT1–SIRT7), are NAD^+^-dependent enzymes that modulate stress responses, mitochondrial function, and aging-related pathways. Several sirtuins, particularly SIRT1 and SIRT3, have been implicated in neuroprotection and metabolic regulation in the brain. Finally, class IV is represented solely by HDAC11, which exhibits unique features overlapping with classes I and II and has emerging roles in immune regulation and energy metabolism [7]. Collectively, these HDAC isoforms are widely expressed throughout the central nervous system, where they contribute to neurodevelopment, synaptic homeostasis, and responses to injury and disease. Their specific distribution and functions in the human brain will be explored in detail in the following section.

## 3. HDAC in the Central Nervous System

**Figure 1:**
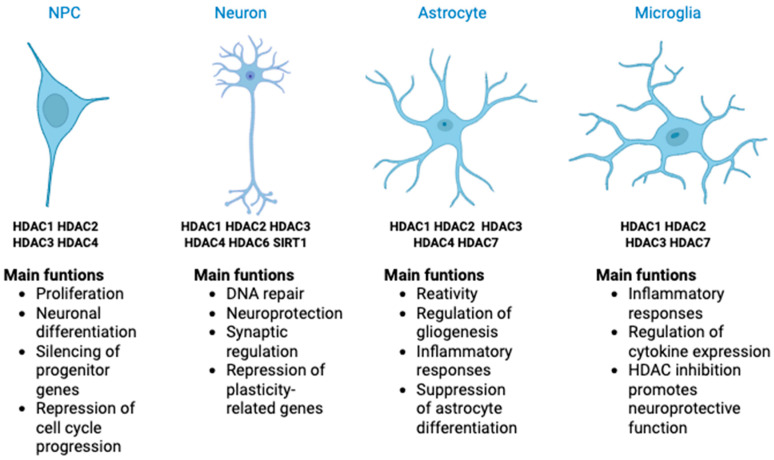
Expression patterns and major functions of HDACs in CNS cell types. This schematic summarizes the predominant HDAC isoforms expressed in neural progenitor cells (NPCs), neurons, astrocytes, and microglia, along with their primary functions. HDACs regulate essential processes such as proliferation, differentiation, synaptic function, gliogenesis, inflammation, and neuroprotection. Their expression is cell-type-specific and dynamically regulated throughout development and in response to injury or disease.

In this section, we discuss the main functions, expression patterns, and inhibition profiles of HDACs across key cell types of the CNS, including neural progenitor cells (NPCs), neurons, and glial cells. HDACs exhibit cell-type and stage-specific roles during development, maturation, and in response to injury or disease. Their modulation through selective or broad-spectrum inhibitors has emerged as a promising therapeutic avenue for various neurodevelopmental and neurodegenerative conditions. Figure 1 provides a schematic summary of the major HDAC isoforms expressed in each CNS cell type and highlights their most relevant functions.

### 3.1. Role of HDACs in Neural Progenitor Cells

Accumulating evidence has shown that HDACs play an important role in various models of neural development, neurodevelopmental disorders, and NDDs, although the underlying mechanisms are not yet fully understood. In this review, we summarize the current understanding of the expression patterns, functions, and regulatory mechanisms of HDACs in neural cells. In the CNS, HDACs are expressed in various cell types, including neurons, astrocytes, oligodendrocytes, and microglia [8]. The differential expression and function of HDACs in these cells contribute to their distinct roles in neurodevelopment, synaptic plasticity, and responses to injury and disease.

HDAC1 is prominently expressed in neural stem cells, progenitors, and glial cells, whereas HDAC2 is mainly found in neural progenitors, post-mitotic neuroblasts, and neurons, but not in fully differentiated glial cells. These findings point to distinct stages of neural development where HDAC1 and HDAC2 play critical roles, suggesting that these deacetylases may regulate key transitions during neural differentiation [9].

Deletion of HDAC1 significantly impairs neuronal differentiation in hippocampal stem cells, and HDAC2 is unable to compensate for this loss. This demonstrates HDAC1’s critical role in neuronal differentiation, which is important for understanding brain development and potential treatments for neurodevelopmental disorders [10].

HDAC activity is crucial for the proper segregation of progenitor cell domains in the developing CNS, specifically between the Nkx2.2 and Olig2 domains. The transcription factor Tcf7l2 (Tcf4) relies on HDAC activity to repress target genes such as Nkx2.2 during CNS development. This Tcf4 repressor complex involves interactions between HDAC and the Gro/TLE/Grg co-repressor [11].

As neural stem cells and progenitors differentiate into neurons in the subventricular zone and dentate gyrus, they switch between HDAC1 and HDAC2 expression. Administration of HDACi like valproic acid (VPA) during specific postnatal periods significantly decreases neural stem cell progeny production in the dentate gyrus, rostral migratory stream, and olfactory bulb. Short-term treatment with HDAC inhibitors such as VPA and trichostatin A (TSA) directly reduces the number and size of neurospheres, which are used to measure neural stem cell activity in vitro [12].

Simultaneous deletion of HDAC1 and HDAC2 in the CNS leads to severe brain development abnormalities, including hippocampal, cerebellar, and cortical defects, resulting in lethality by postnatal day 7. While the deletion of either HDAC1 or HDAC2 alone does not have a noticeable effect, their combined deletion blocks neuronal differentiation and induces excessive cell death. Notably, in vitro experiments show that HDAC1 and HDAC2 deletion halts neuronal differentiation in cortical neuronal precursors, while astrocytic differentiation remains unaffected [13].

Depleting HDAC1 and HDAC2 in radial glial progenitors disrupts the positioning of intermediate progenitors (IPs) at the ventricular surface, causing premature differentiation into neurons and leading to cortical malformations. HDAC1 and HDAC2 regulate IP positioning through the proneural gene Neurogenin2, which plays a critical role in establishing the subventricular zone microenvironment essential for proper cortical development [14].

Deletion of HDAC1 and HDAC2 in neural cells results in severely impaired brain development, leading to reduced proliferation, increased DNA damage, and apoptosis, ultimately causing loss of most brain tissue by embryonic day 18.5. However, the expression of a single allele of HDAC2 is enough to prevent this severe phenotype, while a single allele of HDAC1 only delays the perinatal lethality. The overexpression of the PKCδ gene, a direct target of HDAC2, contributes to the observed impaired brain development and reduced proliferation in Hdac1 Δ/+n Hdac2 Δ/Δn mice [15].

HDAC2 is particularly important for the proper maturation of neurons derived from adult neurogenesis. Its absence leads to increased proliferation and continued expression of progenitor markers in differentiating neurons, indicating that HDAC2 is necessary to silence progenitor genes during adult-generated neuron differentiation. This points to a cell-autonomous function of HDAC2 in adult neurogenesis, highlighting differences in the regulation of neurogenesis between development and adulthood [16].

The absence of HDAC3, unlike HDAC2, initiates a neuronal differentiation pathway in neural stem cells. Removing the corepressor NCOR or HDAC2, along with thyroid hormone T3 treatment, increases the expression of oligodendrocyte genes such as Sox8 and Sox10. Sox10 is crucial for maintaining the differentiated state of neural stem cells by repressing stem cell programming factors like Sox2 and Sox9 [17].

Studies in vertebrate models have been crucial for understanding the role of HDACs in the process of cellular differentiation within the nervous system. In the developing Xenopus, most proliferative precursor cells are radial glial cells, and their numbers decrease with development. HDAC1 regulates the proliferation of radial glial cells, and its knockdown reduces their proliferation. Visual deprivation, which induces radial glial cell proliferation, is mediated by HDAC1, emphasizing its role in regulating glial cell dynamics during development [18]. In zebrafish, HDAC1 is essential for specifying oligodendrocytes, the myelinating cells of the CNS. In the absence of HDAC1, the expression of key transcription factors involved in oligodendrocyte specification, such as olig2, nkx2.2a, and sox10, is disrupted. This demonstrates HDAC1’s critical role in regulating the specification of oligodendrocyte fate from neural progenitors [19].

HDAC3 is another important isoform in neurons and progenitor cells, where it functions as a repressor of synaptic plasticity-related genes [20]. HDAC3 also plays a key role in the proliferation of adult neural stem/progenitor cells by regulating the G2/M phase of the cell cycle through the stabilization of the cell cycle protein CDK1. Modulating HDAC3 activity may be beneficial for tissue regeneration and cancer therapy [21]. Finally, inactivation of the HDAC3 gene leads to developmental defects in the neocortex, hippocampus, and corpus callosum, causing post-weaning lethality and abnormal behaviors such as hyperactivity and anxiety. These defects stem from the rapid loss of embryonic neural stem and progenitor cells, along with premature neurogenesis and abnormal neuronal migration. Cerebral cortices with HDAC3 inactivation display increased DNA damage responses, apoptosis, and histone hyperacetylation, while neural stem and progenitor cells lacking HDAC3 exhibit impaired neurosphere formation in vitro [22].

The inhibition of HDACs leads to region-specific effects on neurogenesis, with HDACs promoting neurogenesis in the ganglionic eminences by downregulating BMP2/4 signaling while inhibiting neurogenesis in the cortex through a similar pathway. This highlights the dual role HDACs play in different brain regions, influencing neural development [23]. Inhibition of HDAC activity, such as through TSA, has been shown to block proliferation, increase neuronal differentiation, and decrease astrocyte differentiation in NPCs. Interestingly, TSA inhibition of HDACs does not affect NPCs’ survival or migration. These effects were linked to a reduction in the expression of class II HDACs, while class I HDACs remained unaffected [24].

Given the distinct roles of HDAC1 and HDAC2 in neural development, the use of selective inhibitors targeting these isoforms, as well as pan-HDAC inhibitors, during early postnatal stages should be approached with caution. Inhibition at this critical period may disrupt the finely tuned epigenetic regulation required for neural stem cell differentiation, neurogenesis, and proper maturation of neuronal circuits, potentially leading to long-lasting structural and functional impairments in the brain.

### 3.2. Role of HDAC in Neuron Cells

HDACs play a pivotal role in the neuronal response to injury, governing both transcription-dependent and transcription-independent processes. This regulation extends beyond generic mechanisms, with individual HDAC isoforms contributing distinctly to neuronal development and repair following injury. By focusing on these individual roles, recent studies have moved beyond traditional broad-spectrum HDAC inhibitors, uncovering more specific therapeutic targets for neurodegeneration [25].

The NuRD complex (Nucleosome Remodeling and Deacetylase complex) is a multi-protein complex that integrates chromatin remodeling and histone deacetylase activities, playing a key role in regulating gene expression, DNA repair, and cellular differentiation by modifying chromatin structure [26]. One such key regulatory mechanism involves the NuRD complex, which contains HDACs and is essential for controlling the expression of synaptogenesis-related genes in the cerebellum. This regulation leads to enhanced presynaptic differentiation, and through participation in the NuRD complex, HDACs in neurons gain functional specificity [27]. In the context of neurotoxicity, the p25/Cdk5 complex has been found to deregulate HDAC1 activity, leading to aberrant cell-cycle activation and DNA damage, which ultimately results in neuronal death. This pathway has been elucidated in transgenic models, where inhibition of HDAC1 by the p25/Cdk5 complex was identified as the underlying mechanism of cell damage. Interestingly, increasing HDAC1 activity counteracts these deleterious effects, offering protection against DNA damage and subsequent neurotoxicity [28].

HDAC inhibition has emerged as a potential neuroprotective strategy, offering defense against neurodegeneration. Numerous studies in experimental models of NDDs show that HDACi can be effective, with their beneficial effects possibly mediated through both epigenetic and non-epigenetic pathways [29]. However, the essential role of HDAC activity in brain development is underscored by the finding that deletion of both HDAC1 and HDAC2 in the CNS results in severe abnormalities, including hippocampal, cerebellar, and cortical defects, and lethality by postnatal day 7. This highlights the redundant yet critical functions of these proteins, as deletion of either HDAC1 or HDAC2 alone has no observable effect. Therefore, selective or cell-type-specific inhibition, rather than broad HDAC suppression, is likely necessary to achieve therapeutic benefit while preserving physiological functions.

HDAC6, specifically, plays a significant role in maintaining synaptic function. In healthy neurons, HDAC6 is localized to the cytoplasm, where it regulates synaptic function [30]. However, under pathological conditions, it translocates to the nucleus, impairing synaptic function and reducing brain-derived neurotrophic factor (BDNF) expression. This impaired function has been linked to cognitive decline in both animal models of multiple sclerosis and human patients. Notably, inhibiting or knocking down HDAC6 can reverse the detrimental effects of acute stress on synaptic function, which includes altered glutamatergic transmission and receptor trafficking [31].

SIRT1, a member of the sirtuin family of deacetylases, is another key player in neuronal DNA repair mechanisms. In response to DNA double-strand breaks, SIRT1 is rapidly recruited to the damage sites, where it interacts with and activates the ATM protein. This activation stabilizes ATM at the sites of damage and, crucially, SIRT1 also binds to and deacetylates HDAC1, enhancing its activity. This process is vital for the repair of DNA through the non-homologous end-joining pathway, highlighting the intersection of deacetylase activity in maintaining neuronal genomic integrity [32].

Interestingly, the inhibition of HDACs has differential effects on neurogenesis across regions of the brain. In the ganglionic eminences, HDACs promote neurogenesis by downregulating BMP2/4 signaling, whereas in the cortex, HDACs inhibit neurogenesis through the same mechanism [23].

Finally, HDAC4 is recognized for its neuroprotective properties, which operate independently of other protective proteins like HDRP. HDAC4 represses CDK1 activity and inhibits cell cycle progression, providing a safeguard against neuronal loss. Mice lacking HDAC4 exhibit elevated CDK1 activity, leading to cerebellar abnormalities and the loss of Purkinje neurons, further demonstrating the essential role of HDAC4 in neuronal health [33].

### 3.3. Role of HDAC in Glial Cells

HDAC2 and HDAC4 are highly expressed in astrocytes, where they regulate the expression of genes involved in gliogenesis and astrocyte reactivity [34].

HDAC3 is also expressed in astrocytes and has been linked to the regulation of astrocyte-mediated inflammatory responses [35]. HDAC3 functions as a molecular switch that regulates the balance between oligodendrocyte and astrocyte lineage commitment. Its deletion leads to an increased production of astrocytes at the expense of oligodendrocytes, with the excess astrocytes originating from oligodendrocyte progenitor cells. HDAC3 achieves this by interacting with p300 to activate genes related to oligodendrocyte differentiation while repressing astrocyte differentiation genes, such as NFIA. Furthermore, HDAC3 modulates the acetylation of Stat3 and competes with it for p300 binding, thus antagonizing astrocyte formation [36].

Similarly, HDAC7 is upregulated in astrocytes in response to inflammatory stimuli such as LPS. It activates the IKK/NF-κB signaling pathway by deacetylating and activating the IKK complex. The upregulation of HDAC7 in astrocytes triggers NF-κB activation, leading to inflammation and anxiety-like behaviors, while the downregulation of HDAC7 reverses these effects [37].

In glioma cells, strong nuclear expression of HDAC1, HDAC2, and NCOR2 has been observed, while NCOR1 and HDAC3 show weaker expression. Interestingly, higher HDAC3 expression is associated with lower tumor grade and better patient survival. Conversely, the expression of HDAC1 and HDAC2 increases with tumor progression and recurrence, with no significant changes in other antigens based on tumor grade [38].

Moreover, class II and IV HDAC mRNA levels are downregulated in glioblastomas compared to low-grade astrocytomas and normal brain tissue. Protein levels of class II HDAC9 are also lower in high-grade astrocytomas. Furthermore, histone H3 is more acetylated in glioblastomas than in normal brain tissue, indicating a disruption in histone modification balance [39].

Nuclear Receptor Corepressors, specifically NCOR1 and NCOR2 (also known as SMRT), are key proteins involved in gene regulation. They repress the activity of nuclear hormone receptors by recruiting epigenetic modifiers, such as HDACs [40]. Additionally, the strong nuclear expression of HDAC1, HDAC2, and NCOR2 in glioma cells, along with weak expression of NCOR1 and HDAC3, suggests a potential role for these proteins in tumor progression and as markers for patient prognosis [38].

HDAC inhibitors are also noted for their role in suppressing the expression of innate antiviral genes, including IFNβ and interferon-stimulated genes, as well as proteins involved in TLR3/TLR4 signaling in human microglia and astrocytes. HDACi suppress the expression of cytokine and chemokine genes in microglia and astrocytes, although with varying effects on different cytokine groups [41]. Furthermore, HDACi strongly suppress the expression and release of LPS-induced cytokines by microglia, suppressing both M1 and M2 activation markers and attenuating microglial migratory behavior. This suggests that HDACi can play a role in suppressing innate immune activation in microglia [42].

In primary human astrocytes and astrocytoma cells, HDAC inhibition reduces GFAP expression while increasing the ratio of the alternatively spliced GFAP isoform GFAPδ to the canonical GFAPα. This inhibition leads to a decrease in histone acetylation at the GFAP promoter, which correlates with reduced GFAP transcription. The altered GFAPδ α ratio results in aggregation of the GFAP network, affecting the intermediate filament network in astrocytes [43].

In microglia, the expression of HDAC1 and HDAC3 increases with aging, correlating with the upregulation of senescence markers in the human hippocampus. These HDACs are particularly enriched in aged microglial populations, suggesting their impact on cellular senescence and inflammatory dysfunctions during the aging process [44]. Microglial phagocytic activity, chemotaxis, and inflammatory cytokine production are also regulated by Tenascin C (Tnc), which induces the expression of HDAC1. Inhibiting HDAC1 reduces Tnc-induced IL-6 and TNF-α production in microglia, suggesting a role for HDAC1 in postnatal microglial function [45].

The selective inhibition of HDAC1 or HDAC2 suppresses the production of inflammatory cytokines like IL-6 and TNF-α in LPS-activated BV2 microglia. These HDACs have redundant roles in regulating the inflammatory response, with HDAC2 compensating for HDAC1 knockdown. The anti-inflammatory effects of HDAC inhibition do not require new protein synthesis, implying that acetylation of non-histone proteins may be involved [46]. Inhibition of HDAC3 further enhances the neuroprotective functions of microglia, boosting phagocytosis and reducing nitric oxide release, which facilitates the microglial response to inflammation [47].

Finally, the prenatal ablation of HDAC1 and HDAC2 impairs microglial development by increasing apoptosis and reducing survival. However, these proteins are not required for adult microglia survival under homeostatic conditions. Deletion of HDAC1 and HDAC2 in microglia (but not other cell types) reduces amyloid load and improves cognitive function in Alzheimer’s disease models by enhancing microglial amyloid phagocytosis [48].

## 4. Implications of HDAC/HDACi in Neurodegenerative Diseases

The most prevalent neurodegenerative disorders today are Alzheimer’s disease (AD) and Parkinson’s disease (PD). In the following section, we will explore the role of histone deacetylases and their pharmacological inhibition in the context of these conditions. Both diseases share overlapping pathogenic mechanisms, including epigenetic dysregulation, glial reactivity, synaptic loss, and neuronal vulnerability. We highlight how specific HDAC isoforms contribute to disease progression and how selective or broad-spectrum HDACi have been investigated as promising therapeutic strategies to counteract these alterations and restore neural homeostasis.

### 4.1. Implications of HDACs and HDACi in Alzheimer’s Disease

Histone acetylation and deacetylation are critical processes in AD, with both increased and decreased acetylation observed in different contexts. HDACi have shown promise in improving memory and synaptic plasticity in AD animal models by regulating histone acetylation. However, the specific patterns of histone acetylation in AD pathology remain unclear, as variations in acetylation are seen across different genes and brain regions [49]. To better illustrate these heterogeneous findings, Table 1 provides an overview of individual HDAC isoforms levels and their reported alterations in AD, organized by brain region.

In AD patients, pronounced changes in the levels of enzymes involved in histone acetylation pathways are observed, particularly in the frontal cortex (F2 area) compared to the hippocampus. This region exhibits dysregulated histone trafficking and catabolism, along with increased histone H3 acetylation levels in cell nuclei, mainly in the frontal cortex [50].

HDAC1 levels are notably reduced in AD patients, which correlates with increased amyloid-β and tau protein levels, brain atrophy, and cognitive decline. This reduction in HDAC I has been observed both in living patients through PET imaging and in post-mortem brain tissue, as well as in an animal model of AD [51]. Additionally, HDAC1 plays a role in DNA repair by modulating the enzyme OGG1 (OGG1 (8-Oxoguanine DNA Glycosylase 1) is an enzyme involved in the base excision repair), to remove oxidative 8-oxoG DNA damage in the brain. Mice lacking HDAC1 experience age-related DNA damage accumulation and cognitive decline, as HDAC1 stimulates OGG1 activity to remove 8-oxoG lesions linked to transcriptional repression [52].

Increased Aβ and HDAC2 protein levels have been reported in AD brains with cell adhesion molecule L1 deficiency compared to AD brains without the deficiency. The recombinant extracellular domain of L1 was shown to reduce HDAC2 mRNA and protein levels in cultured neurons. Furthermore, inhibiting the glucocorticoid receptor prevented the Aβ-induced increase in HDAC2 levels [53].

Elevated HDAC2 levels are also associated with tau hyperphosphorylation, aggregation, and dendritic abnormalities in Alzheimer’s disease. HDAC2 overexpression downregulates miR-101b, which upregulates AMPK. Restoring miR-101b levels or inhibiting AMPK can reverse tau pathology and dendritic abnormalities caused by HDAC2 overexpression, both in vitro and in an Alzheimer’s disease mouse model [54].

Lastly, the transcription factor Sp3 cooperates with HDAC2 to negatively regulate the expression of synaptic genes, thereby impairing synaptic function and plasticity in neurons. Both Sp3 and HDAC2 are elevated in AD patients and mouse models, and knocking down Sp3 improves synaptic dysfunction. Importantly, exogenous expression of an HDAC2 fragment containing the Sp3-binding domain blocked Sp3 from bringing HDAC2 to synaptic genes, which in turn restored synaptic plasticity and memory in a mouse model with severe neurodegeneration [55].

Increased levels of HDAC2 and HDAC3, along with decreased acetyl-H3, have been observed in both AD and mild cognitive impairment models [56]. Supporting these findings, our group recently demonstrated that animals injected with Aβ oligomers exhibited an increase in HDAC activity and a reduction in histone acetylation in the hippocampus, further suggesting the role of this enzyme in regulating neurodegenerative processes [57].

**Table 1:** Specific HDACs and their alterations in AD.

HDAC	Changes in AD	Observed Effects	Brain Region	Reference
HDAC1	↓ Reduced	Correlation with ↑ Aβ and tau, brain atrophy, cognitive decline	General	[51]
HDAC2	↑ Elevated	Tau hyperphosphorylation, aggregation, dendritic abnormalities	General	[53,54,55]
HDAC3	↑ Elevated	Reduced dendritic spine density, neuroinflammation	Hippocampus	[56,58]
HDAC4	↑ Elevated	Associated with amyloid pathology, synaptic dysfunction	Multiple regions	[59,60]
HDAC5	Variable	Loss impairs memory without impacting AD pathogenesis	-	[61]
HDAC6	↑ Elevated	Contributes to neurodegeneration, possible beneficial effects	Cortex, Hippocampus	[62,63,64,65]

HDACi have emerged as an important therapeutic target in AD in recent years. A broader overview of HDACi investigated in AD, including their molecular targets, mechanisms of action, and therapeutic outcomes, is presented in Table 2.

The class I HDACi, tacedinaline, counteracts the disrupted endoplasmic reticulum–mitochondria cross-talk seen in AD models, which includes increased ER-Ca^2+^ retention, mitochondrial Ca^2+^ accumulation, and mitochondrial depolarization. Additionally, tacedinaline reduces the expression of proteins involved in mitochondrial-associated ER membranes and shortens ER–mitochondria contacts in these models [56].

In the hippocampus of APP/PS1 mice, nuclear HDAC3 levels are elevated compared to wild-type controls. HDAC3 inhibition improves spatial memory, reduces amyloid pathology, alleviates neuroinflammation, and increases dendritic spine density in these mice. Conversely, overexpression of HDAC3 worsens these effects, leading to increased Aβ levels, microglia activation, and reduced dendritic spine density [58].

The 3xTg AD mouse model showed a significantly higher uptake of the [18F] TFAHA radiotracer, a marker of HDAC4 expression, in brain regions associated with Alzheimer’s pathology. In a 3D human neural cell model of AD, increased HDAC4 expression and elevated amyloid-beta protein levels were observed. Treatment with an HDAC4 inhibitor upregulated genes related to memory and synaptic plasticity, indicating potential therapeutic benefits of targeting HDAC4 in AD [59]. Modulating HDAC4 may help counteract early dysfunctions in AD, which are characterized by synaptic structural and functional alterations [60].

On the other hand, HDAC5 loss impairs memory function without significantly impacting AD pathogenesis in mouse models. Thus, HDAC5 plays a role in memory consolidation, and future HDAC inhibitor treatments for AD should avoid targeting HDAC5 [61].

HDAC6 expression is notably elevated in the brains of AD patients and animal models. However, whether this overexpression is a cause or consequence of the disease remains unclear. HDAC6’s increased expression may contribute to neurodegeneration in AD, though it might also have some beneficial effects [62].

The newly developed PET probe [18F] PB118 successfully detected elevated HDAC6 levels in AD model mice, particularly in the cortex and hippocampus. This probe may eventually be used to image HDAC6 levels in the human brain, as increased HDAC6 is associated with AD pathology in both animal models and human post-mortem brain tissue. HDAC6 may contribute to the pathogenesis of AD, potentially through mechanisms affecting vulnerable brain regions [63].

Reducing HDAC6 levels improved cognitive deficits in AD mouse models, likely by enhancing mitochondrial trafficking in neurons. This reduction did not impair normal brain function or cognition in healthy mice [64]. Moreover, the newly developed HDAC6 inhibitor PB118 demonstrated multiple mechanisms of action, including clearing amyloid-beta deposits, improving the tubulin/microtubule network, regulating inflammation, and reducing phospho-tau levels in AD models. HDAC6 thus plays a central role in AD pathophysiology and is considered a suitable pharmacological target, though its exact role—whether in amyloid deposition, neuroinflammation, or other neuropathological changes—remains to be clarified [65].

T-518, a potent and selective HDAC6 inhibitor, can cross the blood/brain barrier and inhibit HDAC6 activity when administered orally. In a mouse model of tauopathy, T-518 improved both behavioral and pathological outcomes, highlighting its potential therapeutic effects for AD and related disorders [66].

Finally, compounds 11b and 6a exhibited selective inhibitory activity against different HDAC isoforms, with 11b being more selective for HDAC6 and 6a for class IIa HDACs and HDAC6. These compounds strongly inhibited amyloid-beta aggregation, disrupted Aβ oligomers, and inhibited acetylcholinesterase (AChE) [67].

**Figure 2:**
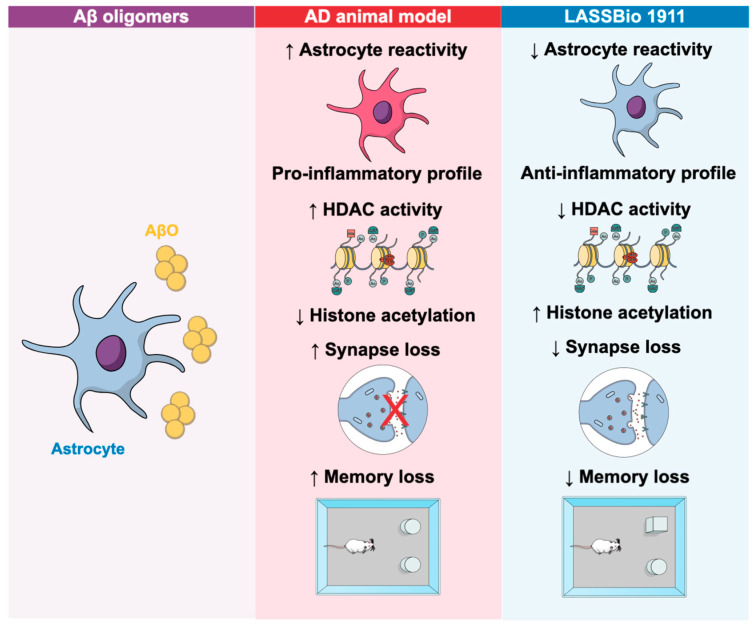
LASSBio-1911 modulates HDAC activity and astrocyte reactivity to reverse synaptic and memory deficits in an AD model. Astrocyte exposure to Aβ oligomers (AβO) induces increased HDAC activity and a pro-inflammatory reactive phenotype in an AD animal model. This leads to reduced histone acetylation, synapse loss, and memory impairment. Treatment with the synthetic compound LASSBio-1911 attenuates astrocyte reactivity, decreases HDAC activity, restores histone acetylation levels, and rescues both synaptic integrity and cognitive function. The schematic summarizes the pathological cascade and therapeutic reversal observed in vivo.

Recently, our group described a new HDAC6 modulator with therapeutic potential for the AD model. We demonstrated that the novel HDAC inhibitor, LASSBio-1911, inhibited HDAC activity and increased acetylated histone levels in the hippocampus of animals infused with amyloid-β oligomers. Moreover, LASSBio-1911 reversed inflammatory cytokine levels, reduced astrocyte reactivity, and enhanced the neuroprotective potential of astrocytes. LASSBio-1911 also rescued cognitive deficits and synaptic loss induced by amyloid-β oligomers (AβO) in mice (Figure 2) [57]. These findings demonstrate its great potential in mitigating the deficits observed in amyloid-β pathology.

HDACi, which restore histone acetylation and transcriptional activation, represent a promising therapeutic approach for NDDs. The dysfunction of HATs and HDACs may serve as a common underlying mechanism contributing to neurodegeneration in both acute and chronic neurological conditions [68]. In summary, reducing HDAC6 levels has been shown to improve cognitive function and synaptic dysfunction in AD models, making HDAC6 inhibition a promising therapeutic strategy for treating cognitive decline in AD. However, no HDAC6 inhibitors have yet been approved by the FDA for AD treatment [62].

The HDACi W2 and I2 reduced levels of AD-associated amyloid-beta (Aβ) peptides in vitro. These inhibitors decreased the expression of enzymes responsible for producing Aβ, while increasing the expression of enzymes that degrade it. Furthermore, the HDACi W2 improved Aβ levels and alleviated learning and memory deficits in a mouse model of AD [69].

Inhibitors of class I histone deacetylases (HDAC1, 2, 3, 8) fully restored contextual memory deficits in a mouse model of AD. The memories consolidated by HDACi-treated transgenic mice remained stable over a two-week period, suggesting that targeted inhibition of class I HDAC isoforms is a promising therapeutic approach for cognitive deficits in early-stage AD [70].

Sodium butyrate, a pan-HDACi, enhanced associative memory in an AD mouse model, even when administered at an advanced stage of pathology. Memory recovery was linked to increased hippocampal histone acetylation and the upregulation of genes associated with associative learning, suggesting that HDACi could offer therapeutic benefits even when administered later in disease progression [71]. In the 5XFAD mouse model of AD, sodium butyrate treatment also improved synaptic plasticity, increased dendritic spine density, and reversed disease-associated reductions in synaptic proteins and increases in pro-inflammatory cytokines [72].

Acetylated histone 4 (H4) levels in the hippocampus of APP/PS1 mice were found to be about 50% lower than in wild-type littermates following fear conditioning. Acute treatment with the HDACi TSA prior to training restored acetylated H4 levels and improved memory performance, as well as long-term potentiation (LTP) in hippocampal slices from these mice [73].

Kainate-induced gamma oscillations were reduced in hippocampal slices from PSAPP AD model mice, compared to controls. However, the HDACi SAHA rescued these gamma oscillation deficits by restoring the activity of fast-spiking interneurons. Interestingly, the effects of SAHA on interneurons differed from those of the acetylcholinesterase inhibitor donepezil, which only restored basal interneuron activity [74].

**Table 2:** HDACi and their therapeutic effects in AD.

Inhibitor	Class/Target	Mechanism of Action	Effects in AD	Model/Study	Reference
Tacedinaline	Class I	Restores ER-mitochondria crosstalk	↓ ER-Ca^2+^ retention, ↓ mitochondrial depolarization	AD models	[56]
SAHA	Pan-HDAC	Restores interneuron activity	Rescues gamma oscillation deficits in hippocampus	PSAPP mice	[74]
BG45	Class I	Modulates GRIP1/AMPA receptor pathway	↑ AMPA subunit expression, ↓ neuronal loss	APP/PS1, APP cells	[75]
TSA	Pan-HDAC	Restores H4 acetylation	Improves memory performance and LTP	APP/PS1	[73]
Sodium butyrate	Pan-HDAC	↑ Hippocampal histone acetylation	Enhances associative memory, ↑ synaptic plasticity	5XFAD, AD models	[71,72]
T-518	HDAC6 selective	Crosses blood/brain barrier	Behavioral and pathological improvements	Tauopathy model	[66]
LASSBio-1911	HDAC6	↑ Histone acetylation	↓ Inflammatory cytokines, ↓ astrocyte reactivity, cognitive rescue	Aβ oligomers	[57]
Compounds 11b/6a	HDAC6/Class IIa	Selective isoform inhibition	↓ Aβ aggregation, ↓ oligomers, AChE inhibition	In Vitro	[67]
W2 and I2	HDACi	↓ Aβ-producing enzymes, ↑ degrading enzymes	↓ Aβ levels, improved learning deficits	AD model	[69]

The class I HDACi BG45 reversed synaptic damage and neuron loss in both APP-transfected cells and APP/PS1 mice by modulating the GRIP1/AMPA receptor pathway. BG45 increased the expression of AMPA receptor subunits (GluA1, GluA2, and GluA3) in APP-transfected cells, while also increasing GRIP1/2 expression and AMPA receptor phosphorylation in APP/PS1 mice. Moreover, BG45 inhibited the expression of HDAC1, HDAC2, and HDAC3, which were elevated in the early stages of the AD models [75].

### 4.2. Implications of HDACs and HDACi in Parkinson’s Disease

Alterations in histone acetylation, an epigenetic mechanism, have been observed in the brains of PD patients. The accumulation of α-synuclein, a hallmark of PD, has been linked to changes in histone acetylation, and modulating this process is being explored as a potential therapeutic approach. Alpha-synuclein directly binds to histones, decreasing the levels of acetylated histone H3 in cultured cells and inhibiting acetylation in histone acetyltransferase assays. Mutations in alpha-synuclein, such as A30P and A53T, which are linked to familial Parkinson’s disease, show increased nuclear localization in cell culture. These findings suggest that nuclear alpha-synuclein plays a role in promoting nigrostriatal degeneration in Parkinson’s disease and support further investigation into histone deacetylase inhibitors as potential therapeutic options for the disorder [76,77].

In recent years, several studies have focused on the role of HDACs in the degeneration of dopaminergic neurons in PD, highlighting the potential of targeting HDACi as a therapeutic strategy [78]. Table 3 illustrates HDAC inhibitors evaluated in Parkinson’s disease, detailing their molecular targets, mechanisms of action, and reported therapeutic effects in experimental and clinical settings.

HDAC6 is highly expressed in Lewy bodies found in PD patients, suggesting it plays a crucial role in the clearance of misfolded and aggregated proteins [79,80]. Although HDAC6 plays an essential role in aggresome formation and the clearance of misfolded proteins, its sustained overactivation in neurodegenerative settings such as Parkinson’s disease may become detrimental. Under chronic proteotoxic stress, excessive HDAC6 activity promotes deacetylation of α-tubulin and other cytoskeletal components, impairing axonal transport, mitochondrial distribution, and synaptic function. It can also modulate chaperone activity, such as that of HSP90, in ways that reduce the efficiency of protein refolding and degradation. Pharmacological inhibition of HDAC6, as with tubastatin A, counteracts these effects by increasing α-tubulin acetylation, improving axonal transport, stabilizing mitochondrial function, and reducing oxidative stress. Thus, while HDAC6 is necessary for aggresome formation, its targeted inhibition in the context of chronic protein misfolding can protect dopaminergic neurons by restoring cellular homeostasis and preventing secondary toxic cascade [81,82].

HDAC6 is also involved in the pathogenesis of α-Synucleinopathies by modulating α-Syn oligomerization and aggregation. Although pathological inclusions such as Lewy bodies (LBs) and glial cytoplasmic inclusions (GCIs) have different structures, they share common components, including HDAC6, indicating that similar protein clearance mechanisms may be involved. However, the exact roles of HDAC6’s enzymatic and non-enzymatic activities in α-Syn inclusion formation remain unclear, necessitating further investigation [83].

In a rat model of PD, the HDACi entinostat improved neurological function and reduced PD markers. Entinostat reversed neurotransmitter imbalances, normalized monoamine oxidase activity, and downregulated the expression of specific HDAC enzymes. Additionally, it reduced PD marker gene expression while increasing neuroprotective gene expression [84].

**Table 3:** HDACi and their therapeutic effects in PD.

Inhibitor	Class/Target	Mechanism of Action	Effects in PD	Model/Study	Reference
Entinostat	Pan-HDAC	Downregulates HDAC enzymes	↓ PD markers, ↑ neuroprotective genes, normalized neurotransmitters	Rat PD model	[84]
NMJ-2/NMJ-3	HDACi (novel)	Reduces oxidative stress and inflammation	↓ Motor/non-motor deficits, restored dopamine levels	MPTP rat model	[85]
Valproic acid	Pan-HDAC	Anti-inflammatory, antioxidant	↓ Motor/non-motor deficits, restored dopamine levels	MPTP rat model	[85]
HGC	HDAC6 selective	Acetylation of NDUFV1 protein	Protected dopaminergic neurons, improved behavior, maintained mitochondrial function	MPTP mouse model	[86]
MC1568	HDAC6 selective	Neuroprotective, anti-inflammatory	Protected dopaminergic neurons, ↓ microglial activation, ↓ forelimb akinesia	6-OHDA rat model	[87]
Tubastatin A	HDAC6 selective	Activates chaperone-mediated autophagy	↑ α-tubulin acetylation, ↓ phosphorylated α-synuclein	Rat PD model	[82]

MPTP administration in rats, a model of PD, induced oxidative stress, inflammation, and dopamine depletion in the striatum. Treatment with novel HDACi NMJ-2 and NMJ-3, as well as the established inhibitor valproic acid, mitigated PD-induced motor and non-motor deficits by reducing oxidative stress and inflammation and restoring dopamine levels. These results suggest HDACi as a rational therapeutic strategy for disease-modifying treatments in PD [85].

The newly synthesized HDAC6i HGC protected dopaminergic neurons from MPP^+^-induced damage in an MPTP-induced PD mouse model. HGC improved behavioral deficits, preserved dopaminergic neurons, and maintained mitochondrial function. The neuroprotective effects of HGC were mediated through the acetylation of the NDUFV1 protein, resulting from HDAC6 inhibition [86].

Peripheral administration of the HDACi MC1568 partially protected against 6-OHDA-induced cell death in cell culture models. In a rat model of PD, MC1568 protected dopaminergic neurons in the substantia nigra and their terminals in the striatum from 6-OHDA-induced neurodegeneration, reducing associated forelimb akinesia. Additionally, MC1568 prevented the increase in microglial activation induced by 6-OHDA in both the striatum and substantia nigra [87].

Selective inhibition of HDAC6 by tubastatin A exhibited protective effects in a rat model of PD. The neuroprotective effects of tubastatin A may result from its ability to activate chaperone-mediated autophagy. Tubastatin A also significantly inhibited the expression of a toxic, phosphorylated form of alpha-synuclein [82].

## 5. Epigenetic Alterations in Human Brain Tissue: HDAC Dysregulation in NDDs

Epigenetic dysregulation has emerged as a key feature in the pathophysiology of neurodegenerative disorders. Among the most studied modifications are histone acetylation and deacetylation, regulated by histone acetyltransferases (HATs) and HDACs. Studies in post-mortem human brain tissues have provided critical insights into how these mechanisms are altered in conditions such as AD, PD, HD, and amyotrophic lateral sclerosis (ALS).

The levels of HDACs were significantly elevated in patients with AD and showed a strong negative correlation with performance across all cognitive assessment tests. Receiver operating characteristic curve analysis demonstrated that HDAC levels exhibited high sensitivity and specificity as potential diagnostic biomarkers [88].

A study mapped genome-wide H3K27ac patterns in the entorhinal cortex of AD patients and controls. It identified over 4000 differentially acetylated regions enriched in AD-related pathways and genes such as *APP*, *PSEN1*, and *MAPT*. These epigenetic alterations were associated with transcriptional changes, revealing key mechanisms involved in AD pathogenesis [89].

In AD, global histone acetylation is reduced, especially at histones H3 and H4, in the hippocampus and temporal cortex, as observed in post-mortem brain tissue [90]. These epigenetic alterations correlate with synaptic dysfunction and cognitive decline. Graff et al. (2012) [91] reported increased HDAC2 expression specifically in hippocampal neurons of AD patients, without changes in HDAC1 or HDAC3 levels, particularly in the CA1 region of patients classified according to Braak and Braak staging. This upregulation of HDAC2 was associated with transcriptional repression of key genes involved in learning and memory, including BDNF and Egr1 [91].

Conversely, higher levels of class I HDAC may exert a neuroprotective role in AD, potentially limiting the accumulation of amyloid-β and tau proteins. Preservation of class I HDAC activity could mitigate the harmful effects of these pathological proteins, helping to prevent brain atrophy and cognitive decline commonly observed in AD [51].

In PD, analyses of post-mortem substantia nigra samples have revealed elevated levels of HDAC1 and HDAC2, accompanied by decreased acetylation of histone H3 at lysine 27 (H3K27ac) in dopaminergic neurons. Using ChIP-seq, Toker et al. (2021) demonstrated a reduction in H3K27ac peaks at promoter regions of genes involved in mitochondrial function and synaptic signaling, suggesting epigenetic repression of neuroprotective pathways [92].

However, increased histone acetylation has also been observed in midbrain dopaminergic neurons of PD patients compared to matched controls [93]. In contrast, other studies have reported reduced HDAC levels in midbrain tissues of PD patients, particularly in experimental models of MPP^+^-induced toxicity and MPTP-treated brains [3].

In Huntington’s disease, there is a specific and significant reduction in the expression of acetylated histones AcH2A, AcH2B, AcH3, and AcH4 in cells of the caudate nucleus and Purkinje cells of the cerebellum, when compared to individuals with frontotemporal lobar degeneration and neurologically healthy controls. Conversely, HDAC5 expression is notably increased in these same regions, suggesting an epigenetic dysregulation associated with histone deacetylation in HD pathology [94]. Significant increases in histone H4 acetylation were observed in post-mortem brains of HD patients. Notably, nuclear acetyl-H4 levels in the HD cortex showed an inverse correlation with mutant huntingtin aggregate load. These results raise concerns about the suitability of HDACi for HD treatment and underscore the need to identify alternative epigenetic targets [95].

A study found no significant differences in HDAC expression levels between patients with ALS and healthy controls, as assessed by Western blotting and reverse-transcription quantitative polymerase chain reaction (RT-qPCR). Consistently, positron emission tomography (PET) imaging with [^11^C] Martinostat, a selective HDAC radiotracer, showed no detectable differences in tracer uptake between ALS participants and control subjects, suggesting the absence of widespread HDAC dysregulation in ALS [96].

ALS is caused by FUS mutations and leads to motor neuron dysfunction characterized by cytoplasmic FUS accumulation, hypoexcitability, and axonal transport defects. These transport defects are reversed by CRISPR/Cas9 correction or by inhibiting histone deacetylase 6, which increases α-tubulin acetylation and ER–mitochondrial interactions. The findings demonstrate that mutant FUS drives pathology and highlight HDAC6 as a potential therapeutic target [97].

Collectively, these findings suggest that aberrant HDAC activity and loss of histone acetylation are common features across NDDs. Such epigenetic signatures not only reflect disease pathology but also support the rationale for using HDACis as potential therapeutic agents. Indeed, restoring histone acetylation in preclinical models has been shown to rescue synaptic plasticity, reduce neuroinflammation, and improve behavior. Nevertheless, translating these observations into clinical practice remains a challenge, due in part to the lack of isoform-selective HDAC inhibitors and the complexity of cell-type-specific epigenetic landscapes in the human brain.

These alterations in HDAC activity and histone acetylation vary across brain regions, cell types, and disease stages. This heterogeneity complicates the use of pan-HDAC inhibitors, as their broad activity could inappropriately target HDAC isoforms that are not contributing to pathology in a given context, leading to off-target effects and unwanted disruptions in gene expression and cellular homeostasis.

Importantly, environmental and lifestyle factors such as physical activity, diet, and stress have been shown to modulate HDAC expression and histone acetylation status. For example, acute aerobic exercise increases histone acetylation in skeletal muscle and influences HDAC activity [98], while mindfulness-based stress reduction is associated with reduced expression of HDAC2, HDAC3, and HDAC9 in peripheral blood cells [99]. Findings suggest that epigenetic plasticity in the adult brain may be influenced by non-pharmacological interventions, offering complementary strategies to slow neurodegenerative progression.

Altogether, the body of evidence supports a unifying model wherein dysregulation of histone acetylation and HDAC activity contributes to synaptic dysfunction, neuronal vulnerability, and cognitive decline across multiple neurodegenerative conditions. Future research should focus on cell-type-specific epigenomic profiling, development of isoform-selective HDACi, and integration of environmental epigenetics to uncover novel therapeutic avenues and improve disease-modifying strategies in neurodegeneration.

## 6. Clinical Trials with HDACi

Although preclinical data on HDACi for the treatment of neurodegenerative diseases are encouraging, their practical application in humans remains incipient. Most initial studies reported in the literature have focused on evaluating these compounds in solid tumors and hematologic cancers. The safety of these agents in the context of neurodegenerative diseases has yet to be established.

Histone deacetylase inhibitors have emerged as promising epigenetic agents in oncology, with multiple clinical trials, from phase I to phase III, conducted to evaluate their safety, pharmacodynamics, and antitumor efficacy across hematologic and solid tumors. HDACi have been extensively studied in the context of tumor biology due to their ability to modulate epigenetic mechanisms involved in cancer progression. By altering chromatin structure and reactivating silenced tumor suppressor genes, HDACi exert antiproliferative, pro-apoptotic, and differentiation-inducing effects on cancer cells. These properties make them attractive candidates for cancer therapy, particularly in hematological malignancies and certain solid tumors.

Below, we present an overview of key clinical trials evaluating various HDAC inhibitors across different phases (Table 4).

AR-42 (also known as OSU-HDAC42) is a phenylbutyrate-derived HDACi with activity against class I and II HDACs. A phase I study of the HDACi AR-42 showed it was safe and may warrant further testing in combination therapies for lymphoma and multiple myeloma. Treatment was associated with a reduction in serum CD44, a biomarker associated with drug resistance in multiple myeloma [100].

SAHA (Suberoylanilide Hydroxamic Acid), also known as vorinostat, is one of the first FDA-approved HDACi. It acts primarily on class I and II HDACs. Another phase I trial of intravenous SAHA confirmed that the drug was well-tolerated and showed antitumor activity. SAHA effectively inhibited its biological target in vivo, evidenced by the accumulation of acetylated histones, and showed efficacy in both solid and hematologic tumors [101]. In another trial, four patients completed at least six cycles of oral SAHA therapy; two had partial responses. The primary side effects were fatigue, dehydration, nausea, and vomiting. These results are encouraging, especially given the limited options for advanced mesothelioma patients after first-line chemotherapy [102].

A phase I trial combining vorinostat with doxorubicin (an anthracycline chemotherapeutic agent) indicated that HDAC2 expression might predict patient response. Antitumor activity was observed in breast, prostate, and melanoma cancers, including two partial responses and two cases of stable disease. HDAC2 levels prior to treatment may serve as a predictive biomarker [103].

Valproic acid, a short-chain fatty acid used primarily as an antiepileptic drug, also functions as a class I HDACi. A clinical and translational phase I trial evaluated the combination of valproic acid (an HDAC inhibitor) with epirubicin (a topoisomerase II inhibitor). The study design was informed by preclinical data and aimed to explore synergistic effects [104].

Depsipeptide, also known as FR901228, is a bicyclic peptide HDACi targeting class I HDACs. Depsipeptide was evaluated in a phase I trial that established the maximum tolerated dose and toxicity profile in patients with refractory neoplasms. Dose-limiting toxicities included fatigue, nausea, vomiting, thrombocytopenia, and cardiac arrhythmia. One patient achieved a partial response without significant cardiac dysfunction [105].

Romidepsin is a selective class I HDACi that primarily targets HDAC1 and HDAC2, with lesser activity against HDAC3 and HDAC8. In a phase II trial, romidepsin as monotherapy for cutaneous T cell lymphoma showed an overall response rate of 34%, including four complete and 20 partial responses. The median response duration was 13.7 months, indicating significant and durable clinical benefit [109]. Romidepsin was ineffective in treating recurrent glioblastoma in a phase I/II trial. The median progression-free survival was only eight weeks, and no patients showed objective responses. Mortality was high, and survival duration was short [111].

LBH589 (Panobinostat) is a potent pan-HDACi that targets class I, II, and IV HDACs. LBH589A, a novel HDACi, was tested in a phase I study assessing its pharmacokinetics and pharmacodynamics in patients with various cancers. A dose-limiting toxicity (prolonged grade 2 thrombocytopenia) was observed at the 7.2 mg/m^2^ dose. Other toxicities included grade 3 neutropenia and hypoglycemia [106].

Pracinostat is an orally bioavailable pan-HDAC inhibitor. The PRIMULA study, a phase III randomized clinical trial, is evaluating its efficacy and safety in combination with azacitidine, a DNA methyltransferase inhibitor, in patients with newly diagnosed acute myeloid leukemia (AML) who are ineligible for intensive chemotherapy. The primary endpoint of the study is overall survival. Preliminary data suggest that the combination does not result in significantly increased toxicity compared to azacitidine alone [107].

Entinostat (MS-275) is a selective class I HDACi. A phase I clinical trial investigated its use in pediatric patients with relapsed or refractory solid tumors, including central nervous system malignancies. The study aimed to establish the maximum tolerated dose, characterize the toxicity profile, and assess pharmacokinetic parameters. Entinostat was well tolerated, with no dose-limiting toxicities (DLTs) observed. Disease progression occurred in all patients within the first two cycles, except for one patient with ependymoma, who achieved stable disease. Based on pharmacokinetic and pharmacodynamic analyses, the recommended phase II dose (RP2D) in pediatric patients was established as 4 mg/m^2^ administered orally once weekly [108].

SB939 (Pracinostat analogue) is a hydroxamic acid-based pan-HDAC inhibitor with oral bioavailability. SB939 was tested in a phase II trial in patients with recurrent/metastatic sarcomas associated with chromosomal translocations. Although no objective responses were observed, 8 out of 14 patients had stable disease, especially those with high HDAC2 expression [110].

Collectively, these studies demonstrate that HDACi are generally well tolerated, with manageable toxicity profiles. Although objective responses are often limited, several agents have shown signs of clinical activity, including disease stabilization and biomarker modulation, supporting their continued development, particularly in combination strategies. Given their epigenetic regulatory properties, HDACi are also promising candidates for the treatment of neurodegenerative diseases, where dysregulated gene expression, neuroinflammation, and mitochondrial dysfunction play central roles.

**Figure 3:**
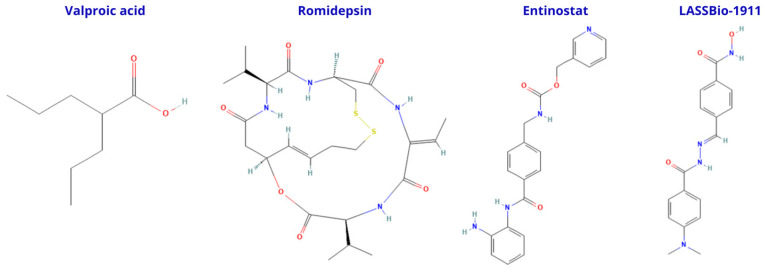
Chemical structures of representative HDACi with different BBB penetration profiles. Valproic acid, a short-chain fatty acid, generally exhibits good BBB permeability but limited isoform selectivity. Romidepsin, a cyclic depsipeptide, is highly potent yet shows poor passive BBB penetration. Entinostat, an aromatic anilide derivative, presents moderate BBB permeability and class I HDAC selectivity. LASSBio-1911 is a synthetic multifunctional compound with HDAC inhibitory and neuroprotective properties; preclinical studies indicate that it inhibits HDAC activity in vivo.

HDACi display marked differences in their ability to cross the blood–brain barrier (BBB), largely determined by their chemical class and physicochemical properties (Figure 3). Small, low-polarity molecules such as short-chain fatty acids, exemplified by valproic acid, tend to penetrate the BBB more efficiently, offering better brain exposure but often with limited isoform selectivity. Cyclic peptides, such as romidepsin, while highly potent, typically show poor passive BBB permeability and may require specialized delivery strategies to reach therapeutic concentrations in the CNS. Aromatic anilide derivatives, such as entinostat, present an intermediate profile, with moderate BBB penetration that can be optimized through structural modifications. Novel synthetic scaffolds, including multifunctional compounds like LASSBio-1911, are being developed to balance potency, selectivity, and CNS accessibility, aiming to overcome the limitations of traditional HDAC inhibitors for neurodegenerative disease treatment.

Future studies should focus on developing CNS-penetrant HDACi, improving pharmacokinetics, and evaluating their efficacy in preclinical and clinical models of AD, PD, and other neurodegenerative conditions. Combination therapies with anti-inflammatory, neuroprotective, or metabolic modulators may further enhance the therapeutic potential of HDAC inhibition in the CNS.

## 7. Conclusions

In conclusion, HDACi have emerged as promising compounds in the treatment of NDDs, offering a novel approach to modulating gene expression and cellular functions. These inhibitors have shown potential not only in altering histone acetylation but also in influencing broader cellular processes, including neuroinflammation, neuronal survival, and synaptic plasticity. By targeting specific cellular pathways, HDACi provide a multifaceted strategy to counteract the progressive nature of neurodegenerative disorders, such as AD and PD.

One of the most exciting prospects lies in the identification of new cellular targets, particularly glial cells, in the context of HDAC inhibition. Glial cells, as key modulators of the brain’s immune response and neuronal health, play an essential role in maintaining homeostasis within the central nervous system. They regulate neuronal activity, BBB integrity, and neuroinflammation, making them critical players in the pathophysiology of neurodegenerative diseases. Targeting glia could offer a new avenue for therapeutic intervention by enhancing neuroprotection and promoting tissue repair in neurodegenerative conditions. Potential strategies to achieve this include the use of nanoparticle-based delivery systems functionalized with glial-specific ligands, antibody–drug conjugates targeting surface markers of reactive astrocytes or microglia, and viral vectors carrying HDACi-related constructs under glial-specific promoters. In addition, defining the optimal therapeutic window for HDACi administration will be crucial, as timing may determine whether these agents modulate glial activity toward a neuroprotective or detrimental phenotype.

Despite the promising potential of HDACi, there are still significant challenges in optimizing their therapeutic efficacy, especially in the context of NDD. One of the primary concerns is the limited ability of these compounds to cross the BBB, which restricts their effectiveness in treating CNS disorders. The BBB acts as a protective barrier, preventing many therapeutic agents from reaching their targets within the brain. Thus, improving the design of HDACi to enhance their permeability across the BBB is a crucial step in their development.

In addition to overcoming the BBB challenge, there is a need to optimize the pharmacokinetic properties of HDACi. This includes enhancing their stability, reducing their metabolism by the liver, and ensuring a controlled and sustained release in the brain. Achieving the right balance between efficacy and safety is vital to ensure that these compounds are not only effective but also well-tolerated in patients. Advances in drug delivery systems, such as the use of nanoparticles, liposomes, or prodrugs, could significantly improve the bioavailability and targeted delivery of HDACi to the CNS.

Moreover, fine-tuning the specificity of these inhibitors to selectively target specific HDAC isoforms is another area that warrants further investigation. Given that HDAC1 and HDAC2 play critical roles in neuronal differentiation, and that HDAC1 expression is reduced in AD, the design of inhibitors that spare or modulate these isoforms in a context-dependent manner becomes particularly important. The broader effects of non-selective HDAC inhibition could lead to unwanted side effects, which may limit their clinical application. Therefore, the development of more selective inhibitors, with the ability to precisely modulate the activity of particular HDAC isoforms involved in neurodegeneration, holds significant therapeutic promise.

In conclusion, while HDACi represent a novel and exciting therapeutic approach for NDDs, there is a pressing need for continued research to refine their molecular design. Improving their ability to cross the BBB, optimizing their pharmacokinetics, and increasing their selectivity will be crucial for realizing the full potential of these compounds in the treatment of CNS disorders.

## Figures and Tables

**Table 4:** Summary of clinical trials with HDACi.

HDACi/Combination	Phase	Cancer Type	Key Findings	Reference
AR-42	Phase I	Lymphoma, Multiple Myeloma	Safe, CD44 reduction	[100]
SAHA (i.v.)	Phase I	Solid and hematologic tumors	Well tolerated, increased histone acetylation	[101]
SAHA (oral)	Phase I	Mesothelioma	Two partial responses; manageable side effects	[102]
Vorinostat + Doxorubicin	Phase I	Breast, Prostate, Melanoma	HDAC2 expression predicts response	[103]
Valproic Acid + Epirubicin	Phase I	Not specified	Preclinical synergy explored	[104]
Depsipeptide	Phase I	Refractory neoplasms	Dose-limiting toxicity observed	[105]
LBH589A	Phase I	Various cancers	Grade 3 neutropenia and hypoglycemia	[106]
Pracinostat + Azacitidine	Phase III	Acute myeloid leukemia	No added toxicity; OS as primary endpoint	[107]
Entinostat	Phase I	Pediatric solid tumors (included CNS)	Maximum tolerated dose determined	[108]
Romidepsin	Phase II	Cutaneous T-cell lymphoma	34% overall response (4 CR, 20 PR); durable benefit	[109]
SB939	Phase II	Sarcomas with translocations	Stable disease in 8/14 patients; ↑ HDAC2 expression	[110]
Romidepsin	Phase I/II	Recurrent glioblastoma	No efficacy; median PFS 8 weeks	[111]

## Data Availability

No new data were created or analyzed in this study.

## Abbreviations

The following abbreviations are used in this manuscript:

Aβ	amyloid-beta peptides
AD	Alzheimer’s disease
ALS	amyotrophic lateral sclerosis
BBB	blood–brain barrier
CNS	central nervous system
GCIs	glial cytoplasmic inclusions
H4	histone 4
HD	Huntington’s disease
HDACs	histone deacetylases
HDACi	histone deacetylase inhibitors
HATs	histone acetyltransferases
LBs	Lewy bodies
MPTP	1-methyl-4-phenyl-1,2,3,6-tetrahydropyridine
NDDs	neurodegenerative diseases
PD	Parkinson’s disease
PET	positron emission tomography

## References

[1] Jiang Q., Liu J., Huang S., Wang X.Y., Chen X., Liu G.H., Ye K., Song W., Masters C.L., Wang J. (2025). Antiageing strategy for neurodegenerative diseases: From mechanisms to clinical advances. Signal Transduct. Target. Ther..

[2] Xiao T., Chen Z., Xie Y., Yang C., Wu J., Gao L. (2024). Histone deacetylase inhibitors: Targeting epigenetic regulation in the treatment of acute leukemia. Ther. Adv. Hematol..

[3] Chuang D.M., Leng Y., Marinova Z., Kim H.J., Chiu C.T. (2009). Multiple roles of HDAC inhibition in neurodegenerative conditions. Trends Neurosci..

[4] Adamu A., Li S., Gao F., Xue G. (2024). The role of neuroinflammation in neurodegenerative diseases: Current understanding and future therapeutic targets. Front. Aging Neurosci..

[5] Milazzo G., Mercatelli D., Di Muzio G., Triboli L., De Rosa P., Perini G., Giorgi F.M. (2020). Histone Deacetylases (HDACs): Evolution, Specificity, Role in Transcriptional Complexes, and Pharmacological Actionability. Genes.

[6] Kumar V., Kundu S., Singh A., Singh S. (2022). Understanding the Role of Histone Deacetylase and their Inhibitors in Neurodegenerative Disorders: Current Targets and Future Perspective. Curr. Neuropharmacol..

[7] Bonomi R.E., Riordan W., Gelovani J.G. (2024). The Structures, Functions, and Roles of Class III HDACs (Sirtuins) in Neuropsychiatric Diseases. Cells.

[8] Han B., Wang M., Li J., Chen Q., Sun N., Yang X., Zhang Q. (2023). Perspectives and new aspects of histone deacetylase inhibitors in the therapy of CNS diseases. Eur. J. Med. Chem..

[9] MacDonald J.L., Roskams A.J. (2008). Histone deacetylases 1 and 2 are expressed at distinct stages of neuro-glial development. Dev. Dyn..

[10] Nieto-Estevez V., Changarathil G., Adeyeye A.O., Coppin M.O., Kassim R.S., Zhu J., Hsieh J. (2021). HDAC1 Regulates Neuronal Differentiation. Front. Mol. Neurosci..

[11] Wang H., Matise M.P. (2016). Tcf7l2/Tcf4 Transcriptional Repressor Function Requires HDAC Activity in the Developing Vertebrate CNS. PLoS ONE.

[12] Foti S.B., Chou A., Moll A.D., Roskams A.J. (2013). HDAC inhibitors dysregulate neural stem cell activity in the postnatal mouse brain. Int. J. Dev. Neurosci..

[13] Montgomery R.L., Hsieh J., Barbosa A.C., Richardson J.A., Olson E.N. (2009). Histone deacetylases 1 and 2 control the progression of neural precursors to neurons during brain development. Proc. Natl. Acad. Sci. USA.

[14] Tang T., Zhang Y., Wang Y., Cai Z., Lu Z., Li L., Huang R., Hagelkruys A., Matthias P., Zhang H. (2019). HDAC1 and HDAC2 Regulate Intermediate Progenitor Positioning to Safeguard Neocortical Development. Neuron.

[15] Hagelkruys A., Lagger S., Krahmer J., Leopoldi A., Artaker M., Pusch O., Zezula J., Weissmann S., Xie Y., Schofer C. (2014). A single allele of Hdac2 but not Hdac1 is sufficient for normal mouse brain development in the absence of its paralog. Development.

[16] Jawerka M., Colak D., Dimou L., Spiller C., Lagger S., Montgomery R.L., Olson E.N., Wurst W., Gottlicher M., Gotz M. (2010). The specific role of histone deacetylase 2 in adult neurogenesis. Neuron Glia Biol..

[17] Castelo-Branco G., Lilja T., Wallenborg K., Falcao A.M., Marques S.C., Gracias A., Solum D., Paap R., Walfridsson J., Teixeira A.I. (2014). Neural stem cell differentiation is dictated by distinct actions of nuclear receptor corepressors and histone deacetylases. Stem Cell Rep..

[18] Tao Y., Ruan H., Guo X., Li L., Shen W. (2015). HDAC1 regulates the proliferation of radial glial cells in the developing Xenopus tectum. PLoS ONE.

[19] Cunliffe V.T., Casaccia-Bonnefil P. (2006). Histone deacetylase 1 is essential for oligodendrocyte specification in the zebrafish CNS. Mech. Dev..

[20] D’Mello S.R. (2020). Histone deacetylase-3: Friend and foe of the brain. Exp. Biol. Med..

[21] Jiang Y., Hsieh J. (2014). HDAC3 controls gap 2/mitosis progression in adult neural stem/progenitor cells by regulating CDK1 levels. Proc. Natl. Acad. Sci. USA.

[22] Li L., Jin J., Yang X.J. (2019). Histone Deacetylase 3 Governs Perinatal Cerebral Development via Neural Stem and Progenitor Cells. iScience.

[23] Shaked M., Weissmuller K., Svoboda H., Hortschansky P., Nishino N., Wolfl S., Tucker K.L. (2008). Histone deacetylases control neurogenesis in embryonic brain by inhibition of BMP2/4 signaling. PLoS ONE.

[24] Liu H., Wu H., Wang Y., Wang Y., Wu X., Ju S., Wang X. (2012). Inhibition of class II histone deacetylase blocks proliferation and promotes neuronal differentiation of the embryonic rat neural progenitor cells. Acta Neurobiol. Exp..

[25] Cho Y., Cavalli V. (2014). HDAC signaling in neuronal development and axon regeneration. Curr. Opin. Neurobiol..

[26] Bornelov S., Reynolds N., Xenophontos M., Gharbi S., Johnstone E., Floyd R., Ralser M., Signolet J., Loos R., Dietmann S. (2018). The Nucleosome Remodeling and Deacetylation Complex Modulates Chromatin Structure at Sites of Active Transcription to Fine-Tune Gene Expression. Mol. Cell.

[27] Sun Y.E., Cheng L., Hu K. (2014). With NuRD, HDACs Go “Nerdy”. Dev. Cell.

[28] Kim D., Frank C.L., Dobbin M.M., Tsunemoto R.K., Tu W., Peng P.L., Guan J.S., Lee B.H., Moy L.Y., Giusti P. (2008). Deregulation of HDAC1 by p25/Cdk5 in neurotoxicity. Neuron.

[29] D’Mello S.R. (2009). Histone deacetylases as targets for the treatment of human neurodegenerative diseases. Drug News Perspect..

[30] Perry S., Kiragasi B., Dickman D., Ray A. (2017). The Role of Histone Deacetylase 6 in Synaptic Plasticity and Memory. Cell Rep..

[31] LoPresti P. (2020). HDAC6 in Diseases of Cognition and of Neurons. Cells.

[32] Dobbin M.M., Madabhushi R., Pan L., Chen Y., Kim D., Gao J., Ahanonu B., Pao P.C., Qiu Y., Zhao Y. (2013). SIRT1 collaborates with ATM and HDAC1 to maintain genomic stability in neurons. Nat. Neurosci..

[33] Majdzadeh N., Wang L., Morrison B.E., Bassel-Duby R., Olson E.N., D’Mello S.R. (2008). HDAC4 inhibits cell-cycle progression and protects neurons from cell death. Dev. Neurobiol..

[34] Dai Y., Wei T., Shen Z., Bei Y., Lin H., Dai H. (2021). Classical HDACs in the regulation of neuroinflammation. Neurochem. Int..

[35] Niu H., Song W., Pei D., Ma C., Liu F., Li Y., Han S. (2022). Depleted histone deacetylase 3 or restored microRNA-19b-1-5p facilitates recovery of spinal cord injury via inactivating JAK2/STAT3 signaling pathway. Genomics.

[36] Zhang L., He X., Liu L., Jiang M., Zhao C., Wang H., He D., Zheng T., Zhou X., Hassan A. (2016). Hdac3 Interaction with p300 Histone Acetyltransferase Regulates the Oligodendrocyte and Astrocyte Lineage Fate Switch. Dev. Cell.

[37] Ye J., Zhong S., Deng Y., Yao X., Liu Q., Wang J.Z., Xiao S. (2022). HDAC7 Activates IKK/NF-kappaB Signaling to Regulate Astrocyte-Mediated Inflammation. Mol. Neurobiol..

[38] Campos B., Bermejo J.L., Han L., Felsberg J., Ahmadi R., Grabe N., Reifenberger G., Unterberg A., Herold-Mende C. (2011). Expression of nuclear receptor corepressors and class I histone deacetylases in astrocytic gliomas. Cancer Sci..

[39] Lucio-Eterovic A.K., Cortez M.A., Valera E.T., Motta F.J., Queiroz R.G., Machado H.R., Carlotti C.G., Neder L., Scrideli C.A., Tone L.G. (2008). Differential expression of 12 histone deacetylase (HDAC) genes in astrocytomas and normal brain tissue: Class II and IV are hypoexpressed in glioblastomas. BMC Cancer.

[40] Ritter M.J., Amano I., Imai N., Soares De Oliveira L., Vella K.R., Hollenberg A.N. (2021). Nuclear Receptor CoRepressors, NCOR1 and SMRT, are required for maintaining systemic metabolic homeostasis. Mol. Metab..

[41] Suh H.S., Choi S., Khattar P., Choi N., Lee S.C. (2010). Histone deacetylase inhibitors suppress the expression of inflammatory and innate immune response genes in human microglia and astrocytes. J. Neuroimmune Pharmacol..

[42] Kannan V., Brouwer N., Hanisch U.K., Regen T., Eggen B.J., Boddeke H.W. (2013). Histone deacetylase inhibitors suppress immune activation in primary mouse microglia. J. Neurosci. Res..

[43] Kanski R., Sneeboer M.A., van Bodegraven E.J., Sluijs J.A., Kropff W., Vermunt M.W., Creyghton M.P., De Filippis L., Vescovi A., Aronica E. (2014). Histone acetylation in astrocytes suppresses GFAP and stimulates a reorganization of the intermediate filament network. J. Cell Sci..

[44] Auzmendi-Iriarte J., Moreno-Cugnon L., Saenz-Antonanzas A., Grassi D., de Pancorbo M.M., Arevalo M.A., Wood I.C., Matheu A. (2022). High levels of HDAC expression correlate with microglial aging. Expert. Opin. Ther. Targets.

[45] Haage V., Elmadany N., Roll L., Faissner A., Gutmann D.H., Semtner M., Kettenmann H. (2019). Tenascin C regulates multiple microglial functions involving TLR4 signaling and HDAC1. Brain Behav. Immun..

[46] Durham B.S., Grigg R., Wood I.C. (2017). Inhibition of histone deacetylase 1 or 2 reduces induced cytokine expression in microglia through a protein synthesis independent mechanism. J. Neurochem..

[47] Meleady L., Towriss M., Kim J., Bacarac V., Dang V., Rowland M.E., Ciernia A.V. (2023). Histone deacetylase 3 regulates microglial function through histone deacetylation. Epigenetics.

[48] Datta M., Staszewski O., Raschi E., Frosch M., Hagemeyer N., Tay T.L., Blank T., Kreutzfeldt M., Merkler D., Ziegler-Waldkirch S. (2018). Histone Deacetylases 1 and 2 Regulate Microglia Function during Development, Homeostasis, and Neurodegeneration in a Context-Dependent Manner. Immunity.

[49] Lu X., Wang L., Yu C., Yu D., Yu G. (2015). Histone Acetylation Modifiers in the Pathogenesis of Alzheimer’s Disease. Front. Cell Neurosci..

[50] Schueller E., Paiva I., Blanc F., Wang X.L., Cassel J.C., Boutillier A.L., Bousiges O. (2020). Dysregulation of histone acetylation pathways in hippocampus and frontal cortex of Alzheimer’s disease patients. Eur. Neuropsychopharmacol..

[51] Pascoal T.A., Chamoun M., Lax E., Wey H.Y., Shin M., Ng K.P., Kang M.S., Mathotaarachchi S., Benedet A.L., Therriault J. (2022). [(11)C]Martinostat PET analysis reveals reduced HDAC I availability in Alzheimer’s disease. Nat. Commun..

[52] Pao P.C., Patnaik D., Watson L.A., Gao F., Pan L., Wang J., Adaikkan C., Penney J., Cam H.P., Huang W.C. (2020). HDAC1 modulates OGG1-initiated oxidative DNA damage repair in the aging brain and Alzheimer’s disease. Nat. Commun..

[53] Hu C., Hu J., Meng X., Zhang H., Shen H., Huang P., Schachner M., Zhao W. (2020). L1CAM Beneficially Inhibits Histone Deacetylase 2 Expression under Conditions of Alzheimer’s Disease. Curr. Alzheimer Res..

[54] Liu D., Tang H., Li X.Y., Deng M.F., Wei N., Wang X., Zhou Y.F., Wang D.Q., Fu P., Wang J.Z. (2017). Targeting the HDAC2/HNF-4A/miR-101b/AMPK Pathway Rescues Tauopathy and Dendritic Abnormalities in Alzheimer’s Disease. Mol. Ther..

[55] Yamakawa H., Cheng J., Penney J., Gao F., Rueda R., Wang J., Yamakawa S., Kritskiy O., Gjoneska E., Tsai L.H. (2017). The Transcription Factor Sp3 Cooperates with HDAC2 to Regulate Synaptic Function and Plasticity in Neurons. Cell Rep..

[56] Marinho D., Ferreira I.L., Lorenzoni R., Cardoso S.M., Santana I., Rego A.C. (2023). Reduction of class I histone deacetylases ameliorates ER-mitochondria cross-talk in Alzheimer’s disease. Aging Cell.

[57] Diniz L.P., Morgado J., Bergamo Araujo A.P., da Silva Antonio L.M., Mota-Araujo H.P., de Sena Murteira Pinheiro P., Sagrillo F.S., Cesar G.V., Ferreira S.T., Figueiredo C.P. (2024). Histone deacetylase inhibition mitigates cognitive deficits and astrocyte dysfunction induced by amyloid-beta (Abeta) oligomers. Br. J. Pharmacol..

[58] Zhu X., Wang S., Yu L., Jin J., Ye X., Liu Y., Xu Y. (2017). HDAC3 negatively regulates spatial memory in a mouse model of Alzheimer’s disease. Aging Cell.

[59] Chen Y.A., Lu C.H., Ke C.C., Chiu S.J., Chang C.W., Yang B.H., Gelovani J.G., Liu R.S. (2021). Evaluation of Class IIa Histone Deacetylases Expression and In Vivo Epigenetic Imaging in a Transgenic Mouse Model of Alzheimer’s Disease. Int. J. Mol. Sci..

[60] Colussi C., Aceto G., Ripoli C., Bertozzi A., Li Puma D.D., Paccosi E., D’Ascenzo M., Grassi C. (2023). Cytoplasmic HDAC4 recovers synaptic function in the 3xTg mouse model of Alzheimer’s disease. Neuropathol. Appl. Neurobiol..

[61] Agis-Balboa R.C., Pavelka Z., Kerimoglu C., Fischer A. (2013). Loss of HDAC5 impairs memory function: Implications for Alzheimer’s disease. J. Alzheimers Dis..

[62] Zhang L., Sheng S., Qin C. (2013). The role of HDAC6 in Alzheimer’s disease. J. Alzheimers Dis..

[63] Bai P., Mondal P., Bagdasarian F.A., Rani N., Liu Y., Gomm A., Tocci D.R., Choi S.H., Wey H.Y., Tanzi R.E. (2022). Development of a potential PET probe for HDAC6 imaging in Alzheimer’s disease. Acta Pharm. Sin. B.

[64] Govindarajan N., Rao P., Burkhardt S., Sananbenesi F., Schluter O.M., Bradke F., Lu J., Fischer A. (2013). Reducing HDAC6 ameliorates cognitive deficits in a mouse model for Alzheimer’s disease. EMBO Mol. Med..

[65] Mondal P., Bai P., Gomm A., Bakiasi G., Lin C.J., Wang Y., Choi S.H., Tanzi R.E., Wang C., Zhang C. (2024). Structure-Based Discovery of A Small Molecule Inhibitor of Histone Deacetylase 6 (HDAC6) that Significantly Reduces Alzheimer’s Disease Neuropathology. Adv. Sci..

[66] Onishi T., Maeda R., Terada M., Sato S., Fujii T., Ito M., Hashikami K., Kawamoto T., Tanaka M. (2021). A novel orally active HDAC6 inhibitor T-518 shows a therapeutic potential for Alzheimer’s disease and tauopathy in mice. Sci. Rep..

[67] Tseng H.J., Lin M.H., Shiao Y.J., Yang Y.C., Chu J.C., Chen C.Y., Chen Y.Y., Lin T.E., Su C.J., Pan S.L. (2020). Synthesis and biological evaluation of acridine-based histone deacetylase inhibitors as multitarget agents against Alzheimer’s disease. Eur. J. Med. Chem..

[68] Langley B., Gensert J.M., Beal M.F., Ratan R.R. (2005). Remodeling chromatin and stress resistance in the central nervous system: Histone deacetylase inhibitors as novel and broadly effective neuroprotective agents. Curr. Drug Targets CNS Neurol. Disord..

[69] Sung Y.M., Lee T., Yoon H., DiBattista A.M., Song J.M., Sohn Y., Moffat E.I., Turner R.S., Jung M., Kim J. (2013). Mercaptoacetamide-based class II HDAC inhibitor lowers Abeta levels and improves learning and memory in a mouse model of Alzheimer’s disease. Exp. Neurol..

[70] Kilgore M., Miller C.A., Fass D.M., Hennig K.M., Haggarty S.J., Sweatt J.D., Rumbaugh G. (2010). Inhibitors of class 1 histone deacetylases reverse contextual memory deficits in a mouse model of Alzheimer’s disease. Neuropsychopharmacology.

[71] Govindarajan N., Agis-Balboa R.C., Walter J., Sananbenesi F., Fischer A. (2011). Sodium butyrate improves memory function in an Alzheimer’s disease mouse model when administered at an advanced stage of disease progression. J. Alzheimers Dis..

[72] Jiang Y., Li K., Li X., Xu L., Yang Z. (2021). Sodium butyrate ameliorates the impairment of synaptic plasticity by inhibiting the neuroinflammation in 5XFAD mice. Chem. Biol. Interact..

[73] Francis Y.I., Fa M., Ashraf H., Zhang H., Staniszewski A., Latchman D.S., Arancio O. (2009). Dysregulation of histone acetylation in the APP/PS1 mouse model of Alzheimer’s disease. J. Alzheimers Dis..

[74] Takasu K., Niidome K., Hasegawa M., Ogawa K. (2021). Histone Deacetylase Inhibitor Improves the Dysfunction of Hippocampal Gamma Oscillations and Fast Spiking Interneurons in Alzheimer’s Disease Model Mice. Front. Mol. Neurosci..

[75] Han Y., Chen L., Liu J., Chen J., Wang C., Guo Y., Yu X., Zhang C., Chu H., Ma H. (2022). A Class I HDAC Inhibitor Rescues Synaptic Damage and Neuron Loss in APP-Transfected Cells and APP/PS1 Mice through the GRIP1/AMPA Pathway. Molecules.

[76] Kontopoulos E., Parvin J.D., Feany M.B. (2006). Alpha-synuclein acts in the nucleus to inhibit histone acetylation and promote neurotoxicity. Hum. Mol. Genet..

[77] Lee J.Y., Kim H., Jo A., Khang R., Park C.H., Park S.J., Kwag E., Shin J.H. (2021). alpha-Synuclein A53T Binds to Transcriptional Adapter 2-Alpha and Blocks Histone H3 Acetylation. Int. J. Mol. Sci..

[78] Mazzocchi M., Collins L.M., Sullivan A.M., O’Keeffe G.W. (2020). The class II histone deacetylases as therapeutic targets for Parkinson’s disease. Neuronal Signal..

[79] Kawaguchi Y., Kovacs J.J., McLaurin A., Vance J.M., Ito A., Yao T.P. (2003). The deacetylase HDAC6 regulates aggresome formation and cell viability in response to misfolded protein stress. Cell.

[80] Miki Y., Mori F., Tanji K., Kakita A., Takahashi H., Wakabayashi K. (2011). Accumulation of histone deacetylase 6, an aggresome-related protein, is specific to Lewy bodies and glial cytoplasmic inclusions. Neuropathology.

[81] Li Y., Gu Z., Lin S., Chen L., Dzreyan V., Eid M., Demyanenko S., He B. (2022). Histone Deacetylases as Epigenetic Targets for Treating Parkinson’s Disease. Brain Sci..

[82] Francelle L., Outeiro T.F., Rappold G.A. (2020). Inhibition of HDAC6 activity protects dopaminergic neurons from alpha-synuclein toxicity. Sci. Rep..

[83] Lemos M., Stefanova N. (2020). Histone Deacetylase 6 and the Disease Mechanisms of alpha-Synucleinopathies. Front. Synaptic Neurosci..

[84] Li H., Shi G., Zha H., Zheng L., Luo Z., Wang Y. (2024). Inhibition of histone deacetylase promotes a neuroprotective mechanism in an experimental model of Parkinson’s disease. Arch. Med. Sci..

[85] Meka S.T., Bojja S.L., Kumar G., Birangal S.R., Rao C.M. (2023). Novel HDAC inhibitors provide neuroprotection in MPTP-induced Parkinson’s disease model of rats. Eur. J. Pharmacol..

[86] Li B., Yang Y., Wang Y., Zhang J., Ding J., Liu X., Jin Y., Lian B., Ling Y., Sun C. (2021). Acetylation of NDUFV1 induced by a newly synthesized HDAC6 inhibitor HGC rescues dopaminergic neuron loss in Parkinson models. iScience.

[87] Mazzocchi M., Goulding S.R., Morales-Prieto N., Foley T., Collins L.M., Sullivan A.M., O’Keeffe G.W. (2022). Peripheral administration of the Class-IIa HDAC inhibitor MC1568 partially protects against nigrostriatal neurodegeneration in the striatal 6-OHDA rat model of Parkinson’s disease. Brain Behav. Immun..

[88] Alsadany M.A., Shehata H.H., Mohamad M.I., Mahfouz R.G. (2013). Histone deacetylases enzyme, copper, and IL-8 levels in patients with Alzheimer’s disease. Am. J. Alzheimers Dis. Other Demen..

[89] Marzi S.J., Leung S.K., Ribarska T., Hannon E., Smith A.R., Pishva E., Poschmann J., Moore K., Troakes C., Al-Sarraj S. (2018). A histone acetylome-wide association study of Alzheimer’s disease identifies disease-associated H3K27ac differences in the entorhinal cortex. Nat. Neurosci..

[90] Santana D.A., Smith M.A.C., Chen E.S. (2023). Histone Modifications in Alzheimer’s Disease. Genes.

[91] Graff J., Rei D., Guan J.S., Wang W.Y., Seo J., Hennig K.M., Nieland T.J., Fass D.M., Kao P.F., Kahn M. (2012). An epigenetic blockade of cognitive functions in the neurodegenerating brain. Nature.

[92] Toker L., Tran G.T., Sundaresan J., Tysnes O.B., Alves G., Haugarvoll K., Nido G.S., Dolle C., Tzoulis C. (2021). Genome-wide histone acetylation analysis reveals altered transcriptional regulation in the Parkinson’s disease brain. Mol. Neurodegener..

[93] Basavarajappa B.S., Subbanna S. (2024). Unlocking the epigenetic symphony: Histone acetylation’s impact on neurobehavioral change in neurodegenerative disorders. Epigenomics.

[94] Yeh H.H., Young D., Gelovani J.G., Robinson A., Davidson Y., Herholz K., Mann D.M. (2013). Histone deacetylase class II and acetylated core histone immunohistochemistry in human brains with Huntington’s disease. Brain Res..

[95] Narayan P., Reid S., Scotter E.L., McGregor A.L., Mehrabi N.F., Singh-Bains M.K., Glass M., Faull R.L.M., Snell R.G., Dragunow M. (2020). Inconsistencies in histone acetylation patterns among different HD model systems and HD post-mortem brains. Neurobiol. Dis..

[96] Dios A.M., Babu S., Granucci E.J., Mueller K.A., Mills A.N., Alshikho M.J., Zurcher N.R., Cernasov P., Gilbert T.M., Glass J.D. (2019). Class I and II histone deacetylase expression is not altered in human amyotrophic lateral sclerosis: Neuropathological and positron emission tomography molecular neuroimaging evidence. Muscle Nerve.

[97] Guo W., Naujock M., Fumagalli L., Vandoorne T., Baatsen P., Boon R., Ordovas L., Patel A., Welters M., Vanwelden T. (2017). HDAC6 inhibition reverses axonal transport defects in motor neurons derived from FUS-ALS patients. Nat. Commun..

[98] Saleem A., Safdar A. (2010). Exercise-induced histone acetylation—Playing tag with the genome. J. Physiol..

[99] Kaliman P., Alvarez-Lopez M.J., Cosin-Tomas M., Rosenkranz M.A., Lutz A., Davidson R.J. (2014). Rapid changes in histone deacetylases and inflammatory gene expression in expert meditators. Psychoneuroendocrinology.

[100] Sborov D.W., Canella A., Hade E.M., Mo X., Khountham S., Wang J., Ni W., Poi M., Coss C., Liu Z. (2017). A phase 1 trial of the HDAC inhibitor AR-42 in patients with multiple myeloma and T- and B-cell lymphomas. Leuk. Lymphoma.

[101] Kelly W.K., Richon V.M., O’Connor O., Curley T., MacGregor-Curtelli B., Tong W., Klang M., Schwartz L., Richardson S., Rosa E. (2003). Phase I clinical trial of histone deacetylase inhibitor: Suberoylanilide hydroxamic acid administered intravenously. Clin. Cancer Res..

[102] Krug L.M., Curley T., Schwartz L., Richardson S., Marks P., Chiao J., Kelly W.K. (2006). Potential role of histone deacetylase inhibitors in mesothelioma: Clinical experience with suberoylanilide hydroxamic acid. Clin. Lung Cancer.

[103] Munster P.N., Marchion D., Thomas S., Egorin M., Minton S., Springett G., Lee J.H., Simon G., Chiappori A., Sullivan D. (2009). Phase I trial of vorinostat and doxorubicin in solid tumours: Histone deacetylase 2 expression as a predictive marker. Br. J. Cancer.

[104] Munster P.N., Marchion D.C., Bicaku E., Sullivan P., Beam C., Mahany J.J., Lush R., Sullivan D.M., Daud A. (2005). Phase I trial of the histone deacetylase inhibitor, valproic acid and the topoisomerase II inhibitor, epirubicin: A clinical and translational Study. J. Clin. Oncol..

[105] Sandor V., Bakke S., Robey R.W., Kang M.H., Blagosklonny M.V., Bender J., Brooks R., Piekarz R.L., Tucker E., Figg W.D. (2002). Phase I trial of the histone deacetylase inhibitor, depsipeptide (FR901228, NSC 630176), in patients with refractory neoplasms. Clin. Cancer Res..

[106] Beck J., Fischer T., Rowinsky E., Huber C., Mita M., Atadja P., Peng B., Kwong C., Dugan M., Patnaik A. (2004). Phase I pharmacokinetic and pharmacodynamic study of LBH589: A novel histone deacetylase inhibitor. J. Clin. Oncol..

[107] Garcia-Manero G., Kaźmierczak M., Fong C.Y., Montesinos P., Venditti A., Mappa S., Spezia R., Ades L. (2019). A Phase 3 Randomized Study (PRIMULA) of the Epigenetic Combination of Pracinostat, a Pan-Histone Deacetylase (HDAC) Inhibitor, with Azacitidine (AZA) in Patients with Newly Diagnosed Acute Myeloid Leukemia (AML) Unfit for Standard Intensive Chemotherapy (IC). Blood.

[108] Bukowinski A., Chang B., Reid J.M., Liu X., Minard C.G., Trepel J.B., Lee M.J., Fox E., Weigel B.J. (2021). A phase 1 study of entinostat in children and adolescents with recurrent or refractory solid tumors, including CNS tumors: Trial ADVL1513, Pediatric Early Phase-Clinical Trial Network (PEP-CTN). Pediatr. Blood Cancer.

[109] Piekarz R.L., Frye R., Turner M., Wright J.J., Allen S.L., Kirschbaum M.H., Zain J., Prince H.M., Leonard J.P., Geskin L.J. (2009). Phase II multi-institutional trial of the histone deacetylase inhibitor romidepsin as monotherapy for patients with cutaneous T-cell lymphoma. J. Clin. Oncol..

[110] Chu Q.S., Nielsen T.O., Alcindor T., Gupta A., Endo M., Goytain A., Xu H., Verma S., Tozer R., Knowling M. (2015). A phase II study of SB939, a novel pan-histone deacetylase inhibitor, in patients with translocation-associated recurrent/metastatic sarcomas-NCIC-CTG IND 200dagger. Ann. Oncol..

[111] Iwamoto F.M., Lamborn K.R., Kuhn J.G., Wen P.Y., Yung W.K., Gilbert M.R., Chang S.M., Lieberman F.S., Prados M.D., Fine H.A. (2011). A phase I/II trial of the histone deacetylase inhibitor romidepsin for adults with recurrent malignant glioma: North American Brain Tumor Consortium Study 03-03. Neuro Oncol..

